# Coronavirus-induced coagulopathy during the course of disease

**DOI:** 10.1371/journal.pone.0243409

**Published:** 2020-12-17

**Authors:** Marie Sophie Friedrich, Jan-Dirk Studt, Julia Braun, Donat R. Spahn, Alexander Kaserer

**Affiliations:** 1 Institute of Anesthesiology, University and University Hospital Zurich, Zurich, Switzerland; 2 Division of Medical Oncology and Hematology, University and University Hospital Zurich, Zurich, Switzerland; 3 Departments of Epidemiology and Biostatistics, Epidemiology, Biostatistics and Prevention Institute, University of Zurich, Zurich, Switzerland; Universite de Liege (B34), BELGIUM

## Abstract

**Background:**

A significant proportion of patients with coronavirus disease 19 (COVID-19) suffer from excessive coagulation activation and coagulopathy which is associated with an increased risk of venous and arterial thromboembolism and adverse outcome. Our study investigates coagulation markers and the incidence of thromboembolic events in COVID-19 patients receiving recommended anticoagulation strategies.

**Methods:**

In a retrospective single-center analysis at the University Hospital Zurich, Switzerland, we investigated 31 adult COVID-19 patients between April 6^th^ and May 13^th^, 2020 and with at least one laboratory assessment of the coagulation markers prothrombin time/Quick, thrombin time, fibrinogen and D-dimers. For antithrombotic prophylaxis low-molecular-weight-heparin or unfractionated heparin was administered and two patients with heparin-induced thrombocytopenia received argatroban.

**Results:**

We analyzed 31 patients (68% male, mean age 60± SD 15 years). 22 (71%) of these required intensive care unit treatment, 5 (16%) were hospitalized in a ward, and 4 (13%) were outpatients. Mean fibrinogen levels were markedly elevated to 6.4± SD 1.8g/l, with a peak in the third week of the disease and no significant decrease over time. D-dimers were elevated to a mean value of 5.1±4.4mg/l with peak levels of 6.8±5.3mg/l in the fourth week of disease, and a subsequent decrease. Platelet count (308±136G/l) and PT/Quick (85±22%) showed no significant changes over time. Sensitivity analyses for patients treated in the ICU showed that D-dimer levels were higher in this group. The results of other sensitivity analyses were comparable. Thromboembolic events were diagnosed in 4 (13%) patients and 5 (16%) patients died during the observation period.

**Conclusion:**

We find coagulation alterations in COVID-19 patients indicating significant hypercoagulability. These alterations are visible despite antithrombotic treatment, and peak around week 3–4 of the disease.

## Background

Coronavirus disease 19 (COVID-19) is caused by infection with Severe Acute Respiratory Syndrome Corona Virus-2 (SARS-CoV-2). It was first discovered in December 2019 in Wuhan, China, and has since spread worldwide resulting so far in over 41 million infected persons and over 1 million deaths [1, 2]. Symptoms as fever, cough, shortness of breath, fatigue and pneumonia are described [1, 3]. SARS-CoV-2 is transmitted principally by aerosol. An increasing number of studies indicate that COVID-19 is, however, not only a respiratory disease. SARS-CoV-2 may directly infect endothelial cells thereby causing endotheliitis [4], and COVID-19 is associated with a marked coagulation activation and an increased risk of venous and arterial thromboembolism [5, 6]. To this regard an initial elevation of D-dimers, a marker of coagulation activation and fibrinolysis, is indicative of a poor prognosis [7]. A potential prognostic value of other coagulation parameters is poorly understood. Recognizing their increased risk of thromboembolism, the Swiss Society of Hematology’s (SSH) Working Party Hemostasis has among other national and international societies issued a guideline recommending thromboprophylaxis in all hospitalized COVID-19 patients [8, 9], preferentially with low molecular weight-heparin (LMWH) and with a dose increase if additional prothrombotic risk factors are present. This guideline also suggests daily coagulation monitoring including prothrombin time (PT) / Quick, D-dimers, fibrinogen, and platelet count. It was put into effect in our University Hospital on April 7^th^ and updated on May 25^th^, 2020. Our study investigates laboratory markers of coagulopathy in COVID-19 patients receiving antithrombotic treatment, and the incidence of thromboembolic events.

## Methods

This study was approved and the requirement for written informed consent was waived by the local ethics committee (Kantonale Ethikkommission Zurich, BASEC no. 2020–00849). Data were handled according to the Good Clinical Practice Guidelines. We encrypted our database after data collection was completed. Statistical analysis was performed with encrypted data only.

### Study design

The study is a retrospective single-center study carried out at the University Hospital Zurich, Switzerland. It includes adult patients with confirmed SARS-CoV-2 infection, diagnosed between April 6^th^ and May 13^th^, 2020, and with at least one standardized assessment of coagulation markers.

Standardized coagulation monitoring was recommended on a daily base and by a predefined set of parameters; its execution was, however, left to the attending physicians’ discretion. Patients were not included if they refused to participate, or if standardized coagulation analysis was not available. The study was approved by the local ethics committee.

### Study endpoints

Our primary goal was the investigation of COVID-19 associated coagulopathy during the course of disease, as visualized by a standardized set of coagulation parameters (PT/Quick, thrombin time, fibrinogen, D-dimers and platelet count). In addition, we assessed the incidence of in-hospital thromboembolic events.

### COVID-19 coagulation analyses and anticoagulation strategy

A set of coagulation parameters was predefined according to the SSH recommendations which included PT/Quick, thrombin time, fibrinogen, and D-dimers [8]. Coagulation analyses were performed in platelet-poor citrated plasma using a CS-5100 coagulation analyser (Siemens, Marburg, Germany). Prothrombin time as tissue-factor induced coagulation time was performed using Innovin (Siemens, Marburg, Germany) as a thromboplastin reagent. As it is convention in German-speaking countries, the prothrombin time is displayed as Quick value (% of a normal plasma pool) and as international normalized ratio (INR). Thrombin time was determined using Thromboclotin as thrombin reagent (Siemens, Marburg, Germany). Fibrinogen concentration was determined by functional assay according to *Clauss* (Multifibren U, Siemens, Marburg, Germany). D-dimers were determined by immuno-turbidimetric assay (Innovance D-Dimers, Siemens, Marburg, Germany).

Daily standardized coagulation monitoring was suggested, but execution was left to the attending physicians’ discretion. All hospitalized COVID-19 patients received LMWH or UFH at a prophylactic to intermediate (thromboprophylaxis) or therapeutic (acute thromboembolism, extracorporeal membrane oxygenation [ECMO]) level. Two patients with suspected heparin-induced thrombocytopenia received argatroban. Anti Xa activity (for heparins) and anti IIa activity (for argatroban) was monitored when indicated. Use of direct oral anticoagulants was not documented in this context.

### Variables and data collection

Medical records of all COVID-19 positive patients receiving at least one laboratory COVID-19 coagulation assessment were reviewed. The following parameters were extracted from the hospital’s clinical information system: age, sex, date of initial diagnosis of COVID-19, date and time of blood withdrawal, D-dimers, fibrinogen, PT / Quick, platelet count, thrombin time, anti-factor Xa activity, c-reactive protein (CRP), patient category (ICU, ward, outpatient), duration of hospitalization, outcome (death, discharge, ongoing hospitalization), type of anticoagulant (LMWH, UFH, argatroban), pre-existing comorbidities grouped by organ system (cardiovascular, pulmonal, diabetes, renal, obesity, coagulation disorders, neoplasia), and complications during hospital stay (pulmonary embolism, deep vein thrombosis, acute kidney injury, encephalopathy, cardiovascular complications, and hepatopathy).

### Statistical analysis

Categorical data are reported as frequency and percent and numerical data as mean with standard deviation. To avoid a potential bias of patients with multiple repetitive measurements, the mean value per week and patient was calculated and entered into the figures. Boxplots were used to descriptively show the development of the different coagulation parameters over time. Note that the mean value of each patient per week was entered in these plots so that each patient contributed only one value per week.

To assess the course of the coagulation parameters over time including all longitudinal measurements of the same patients and thus taking into account that these values are not independent, linear mixed models with random intercept per patient were calculated including the day since disease onset as covariate. As the course of the D-dimers suggested a rather quadratic than linear development over time, the day since disease onset was additionally included as quadratic term. In the model for the course of fibrinogen, the natural logarithm of CRP was included in addition to the time variable. An additional model for thrombin time was calculated including UFH, argatroban and type of stay. To take the upper detection limit for fibrinogen, quick and d-dimer into account, a mixed tobit model was applied to analyze these parameters. All statistical analyses were performed with R (Version 3.6.2, R Foundation for Statistical Computing). A p-value of <0.05 was considered statistically significant.

## Results

According to inclusion and exclusion criteria, 31 patients were analyzed. Mean age was 60 ± 15 years, and 68% were male. The most frequent pre-existing comorbidities were cardiovascular, diabetes, and obesity (Table 1). 22 patients (71%) were treated in the ICU and 5 (16%) in the ward, while 4 (13%) were outpatients. In 5 ICU patients (16%) the development of a severe acute respiratory distress syndrome (ARDS) required ECMO treatment. 5 patients (16%) died during the time of observation. The duration of hospitalization is known for 29 patients (48 ± 26 days) while 2 were still hospitalized (Table 1).

**Table 1:** Patient overview (n = 31).

Age (years)		60	± 15
Sex	male	21	68%
Preexisting comorbidities	cardiovascular	18	58%
	pulmonary	9	29%
	diabetes	18	58%
	renal	8	26%
	obesity	10	32%
	coagulation disorder	1	3%
	neoplasia	5	16%
Type of stay	outpatient	4	13%
	ward	5	16%
	ICU	22	71%
ICU patient with ECMO		5	16%
Unfractioned heparin	therapeutic	14	45%
	prophylactic	9	6%
Low molecular heparin	prophylactic	2	6%
Argatroban	therapeutic	2	6%
No anticoagulation		4	13%
Duration of hospitalisation (days, n = 29)		48	± 26
In-hospital mortality		5	16%

Duration of hospitalization is reported for 29 patients, while 2 patients are still hospitalized. 4 outpatients did not receive anticoagulants. Data is presented as count and percentage or mean with standard deviations.

All hospitalized patients received pharmacological antithrombotic prophylaxis or therapy in contrast to none of the outpatients. As antithrombotic drug, UFH was administered in all but 2 patients (6%) who received LMWH. 2 (6%) patients were switched from UFH to argatroban when a heparin-induced thrombocytopenia was suspected (Table 1).

**Fig 1:**
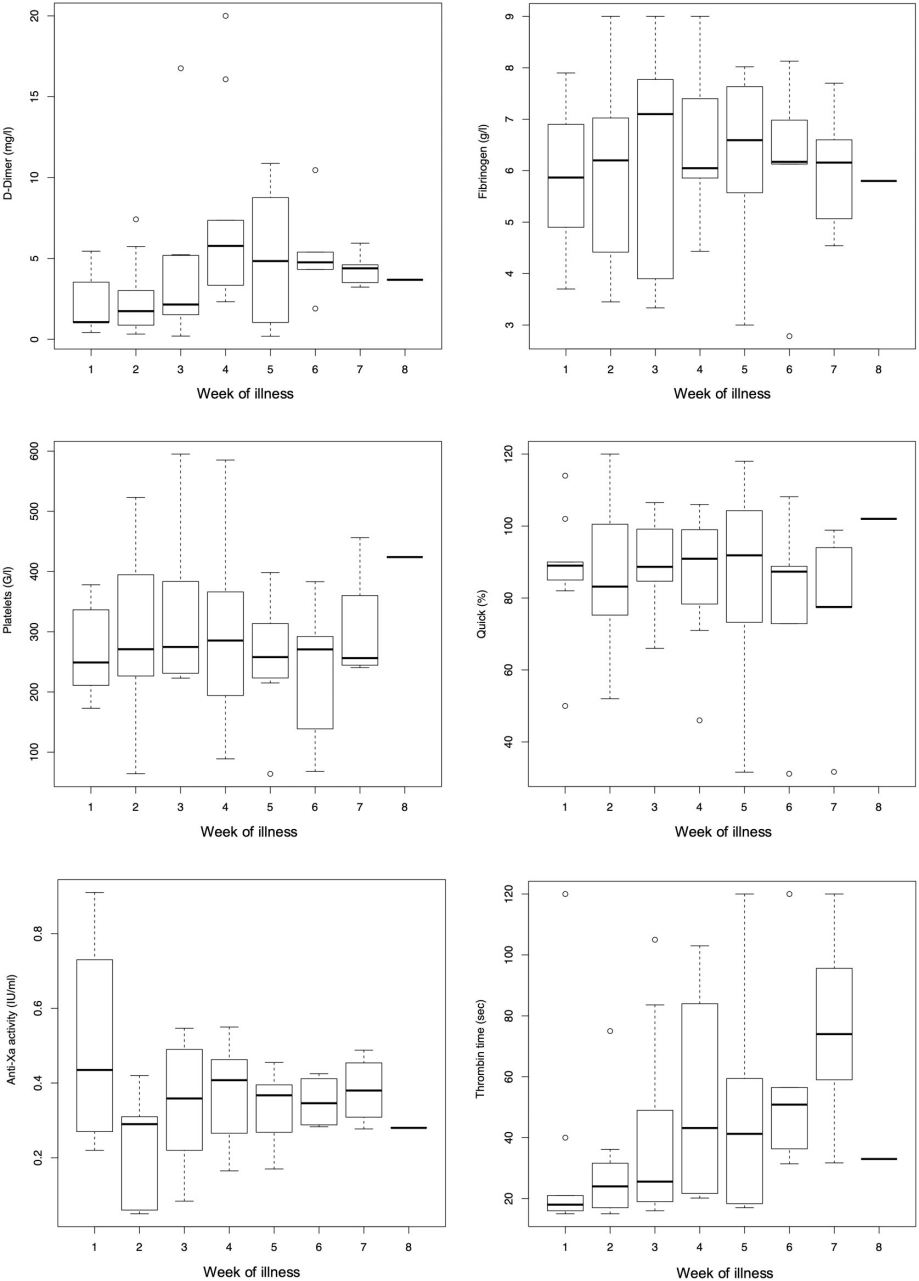
Weekly boxplots of D-dimer, PT/Quick, fibrinogen, thrombin time, anti-Xa activity and platelet count during the course of disease of 31 COVID-19 patients. To avoid a potential bias of patients with multiple repetitive measurements, only the mean value per week and patient was entered in the figures.

Coagulation monitoring showed a hypercoagulable state, despite antithrombotic prophylaxis or therapy in all hospitalized patients. For detailed visualizations of PT/Quick, thrombin time, fibrinogen, D-dimers, and platelet count during the course of disease see Fig 1.

**Fig 2:**
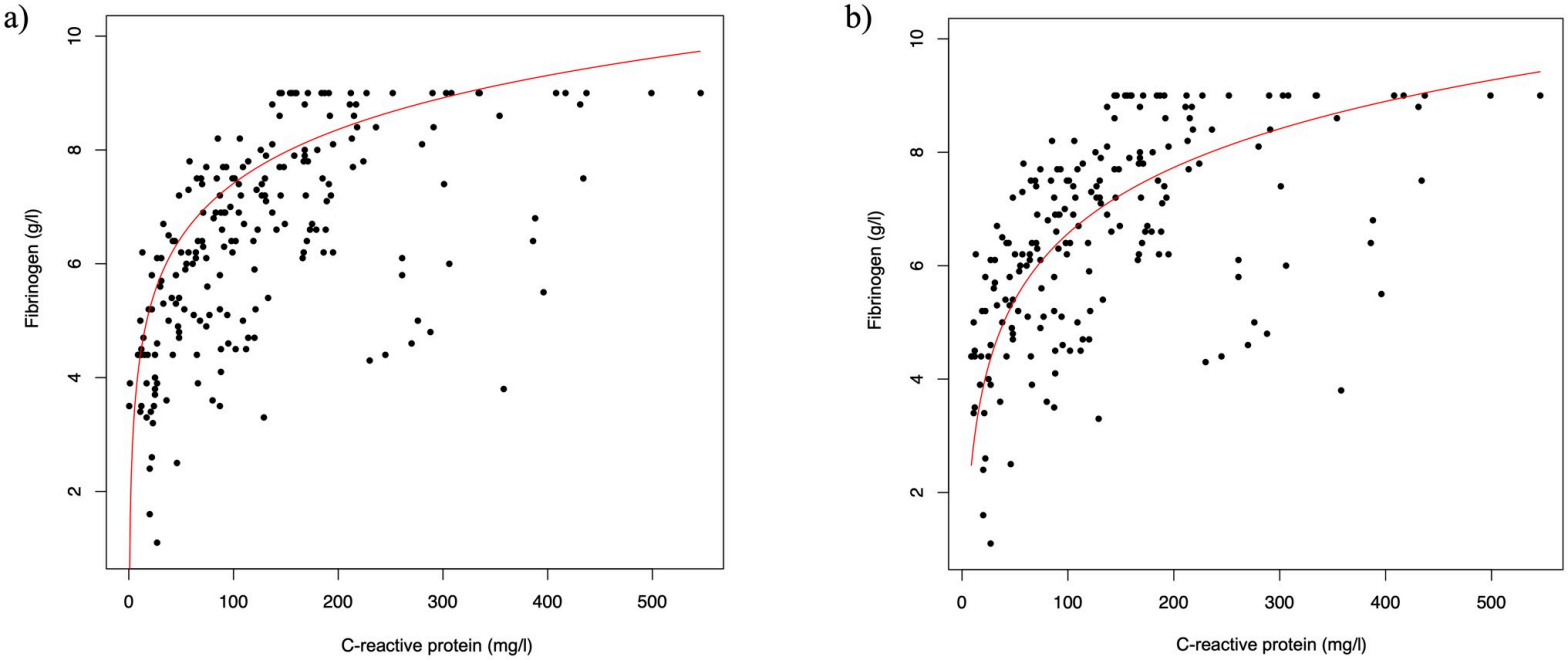
Relation of CRP and fibrinogen levels of 31 COVID-19 patients. The scatterplot on the left (a) shows the analysis of all patients and the graph on the right (b) shows the sensitivity analysis for ICU patients only with comparable results. The red line shows the predicted values from a mixed tobit model including day of illness (here day 27) and log(CRP).

Fibrinogen was markedly elevated to a mean level of 6.4 ± 1.8 g/l, with the highest values in the third week of disease. Also, CRP was elevated to a mean level of 131 ± 106 mg/l. The tobit regression model showed a significant change of fibrinogen over the observation time (-0.02, 95% CI -0.04 to -0.01; p = 0.0051) if no other variables were included and also if adjusted for log(CRP) (0.03, 95% CI 0.01 to 0.05; p = 0.0003). The model additionally shows very strong evidence for an impact of log(CRP) on the fibrinogen levels (1.37, 95% CI 1.17 to 1.57; p<0.0001). Sensitivity analyses for the group of ICU patients showed comparable results (S1 Material). Fig 2 shows a scatterplot of the CRP and fibrinogen values for all patients (left) and for the ICU patients only (right). The red line is the predicted regression line from the mixed tobit regression model and illustrates the relation between the two values.

**Fig 3:**
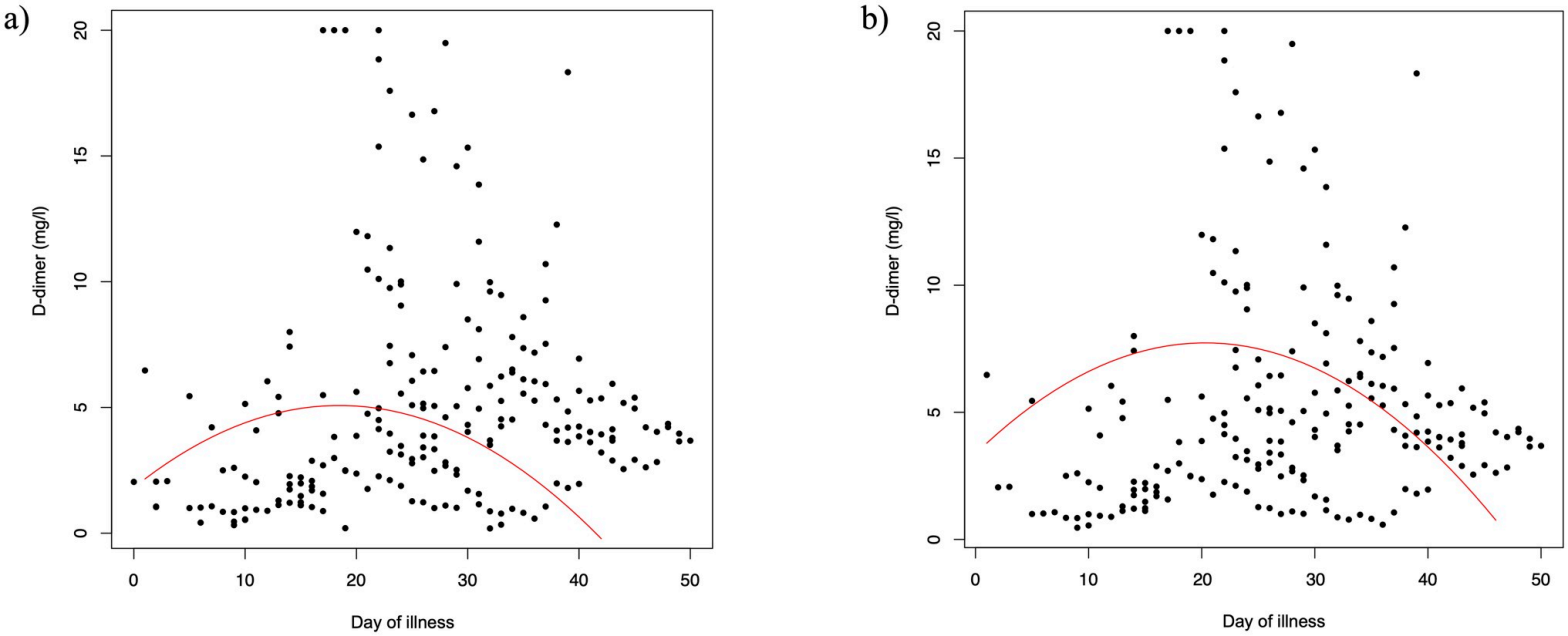
Predicted D-dimer course during the course of disease of 31 COVID-19 patients. The scatterplot on the left (a) shows the analysis of all patients and the graph on the right (b) shows the sensitivity analysis for ICU patients only. The red line shows the predicted D-dimer course obtained from a mixed tobit model, where day of illness is entered both with a linear and a quadratic term. The sensitivity analysis of ICU patients only (b) shows that patients with a severe course of disease may have higher D-dimer levels.

D-dimers were elevated to a mean value of 5.1 ± 4.4 mg/l with a continuous increase over time, peak levels of mean 6.8 ± 5.3 mg/l in the fourth week of disease, and a subsequent decrease. The tobit regression model shows significant changes of D-dimer levels over time (linear coefficient 0.35, 95% CI 0.24 to 0.47; p<0.0001). Fig 3 shows the predicted course over time derived from the tobit regression model for all patients (left) and for ICU patients only (right).

Platelet count and PT/Quick did not show a significant change over time (0.68, 95% CI -1.07 to 2.42, p = 0.44 and 0, 95% CI -0.09 to 0.09; p = 0.96, respectively) (Fig 1).

Fig 1 shows an almost linear increase of the thrombin time over time, confirmed by a regression model indicating a significant increase of 0.66 seconds per disease day (95% CI 0.17 to 1.15, p = 0.008). However, this increase was reduced when anticoagulants were considered as additional explanatory variables in the regression model (S1 Table in S1 Material). The increase of the thrombin time may therefore to the most extent be attributed to an effect of anticoagulation.

During the observation period we diagnosed 5 (16%) new thromboembolic events in 4 (13%) patients. Other complications during that time included acute kidney injury, hepatopathy, cardiac arrythmias, encephalopathy, COVID-19 associated acute myocardial injury and cardiac arrest (Table 2).

**Table 2:** In-hospital complications of COVID-19 patients (n = 31).

Thromboembolic events (all)	5	16%
Pulmonary embolism	1	3%
Deep vein thrombosis	4	13%
Acute kidney injury	20	65%
Encephalopathy	6	19%
Hepatopathy	8	26%
COVID-19 associated acute myocardial injury	4	13%
Cardiac arrhythmia	7	23%
Cardiac arrest requiring CPR	2	6%

Data is presented as count and percentage.

## Discussion

Our retrospective single-center study investigated coagulation markers in COVID-19 patients during the course of disease, and the diagnosis of thromboembolic events during their hospitalization. Our findings confirm a marked procoagulatory state which is observed despite routine antithrombotic treatment and shows a peak around week 3 to 4 of the disease. Sensitivity analyses for ICU patients only showed that these patients may have higher D-dimer levels.

COVID-19 associated alterations of hemostasis is multifactorial including systemic inflammation and activation of the complement system [10, 11]. According to Iba et al. [12], thrombus formation in COVID-19 patients is caused by four major factors: first, COVID-19 induces a cytokine storm which activates coagulation. Second, the fibrinolytic system is suppressed. Third, platelets are activated by the cytokine storm and fourth, endothelial damage induced by inflammation binds platelets and further accelerates the thrombotic reaction. These mechanisms culminate in a hypercoagulability which is a key issue of COVID-19 patients [6, 13–16]. Elevation and rapid increase of D-dimers indicates a procoagulatory state and was also shown to be an independent predictor for thromboembolic events [10, 16, 17]. Elevated D-Dimer levels are not only a marker of coagulation activation and fibrinolysis, they were also shown to be an independent prognostic predictor regarding the in-hospital mortality [1, 18]. D-dimers may therefore be a helpful marker to guide the clinical management of COVID-19 patients [1, 18]. In line with these findings, we find elevated D-dimers already upon hospital admission and observe their further increase with a peak around week 4. Our sensitivity analyses of ICU patients showed that despite antithrombotic treatment these patients may even have higher D-dimers. According to recent guideline recommendations, this could be an argument for an early increase of the heparin dose from a prophylactic to an intermediate level in patients who are critically ill or show additional prothrombotic risk factors [16]. However, D-dimer levels alone are not recommended to guide an individual anticoagulation therapy [10].

Fibrinogen is acting as a mediator of platelet aggregation, red cell adhesion, and thrombosis [19]. It is also an acute phase reactant produced in the liver and fibrinogen levels can therefore be a useful monitoring tool of the inflammatory state [20]. As mentioned above, the infection with coronavirus induces a cytokine storm [11] and interleukin-6 is a major initiator of the acute phase response [21, 22]. Ranucci et al. described a significant association of interleukin-6 with fibrinogen levels [23]. At the beginning of an acute phase reaction fibrinogen’s function seems to be predominant to its role in clot formation [24]. The in the course induced hyperfibrinogenemia has a protective role as part of the host defense against pathogens [24]. However, a prothrombotic risk factor of a high fibrinogen level is still discussed [19, 25, 26]. Accordingly, when correlating CRP and fibrinogen in our study we find an inflammation-induced increased fibrinogen synthesis. Fibrinogen levels peaked in week three of the disease followed by a slow decline but were still markable elevated in week eight. In our opinion the persistent inflammatory state of COVID-19 patients contributes to this prolonged hyperfibrinogenemia. A sensitivity analysis of ICU patients showed no noticeable change in the fibrinogen course.

Platelets also play an important role in the clearance of viral pathogens. A unique feature of COVID-19 is the presence of extramedullary megakaryocytes that actively produce platelets explaining the continuous high platelet count in our study [27]. Moreover, proinflammatory cytokines like interleukin-1β or interleukin-6 also increase platelet production and release [12]. In contrast to our finding it was shown, that COVID-19 patients often suffer from a mild thrombocytopenia due to an increased platelet consumption together with a corresponding increase in platelet production [28]. The lack of thrombocytopenia in our study reflects, that the COVID-19 induced alterations of the hemostasis were not a consumptive coagulopathy—typical of disseminated intravascular coagulation.

Heparin is known to have also anti-inflammatory properties due to binding inflammatory cytokines [29]. Moreover, heparin may prevent viral attachment by binding to host or viral glycoproteins [30]. Several studies have compared the rate of thrombosis in COVID-19 patients with different anticoagulation regimes. Maatman et al. found thromboembolic events in 28% of patients with severe COVID-19 and concluded that a routine thromboembolism prophylaxis may therefore be inadequate [31]. Klok et al. found thrombotic complications in 31% of ICU patients and therefore strongly recommended pharmacological thrombosis prophylaxis in all COVID-19 patients [13]. The high incidence of thromboembolic events suggests an important role of COVID-19-induced coagulopathy [15]. Further studies are needed to investigate its molecular mechanism and the effect of therapeutic interventions [32]. While Al Samkari et al. described a thrombotic complications in 4.8% of COVID-19 patients with standard doses of prophylactic anticoagulation [14], Helms et al. found 16.7% of thrombotic events in their study collective and therefore recommended higher anticoagulation targets, especially in critically ill patients [16]. Compared with these, the incidence of thromboembolism in our patients was lower. This may in part be due to early antithrombotic treatment by guidelines [8, 9]. However, aiming at further reducing this important complication an increase of prophylactic dose heparin to an intermediate level in selected critically ill patients as suggested by revised guidelines may prove beneficial. We observed a linear increase of the thrombin time over time in our patient collective. Our model showed that this increase of the thrombin time may to the most extent be attributed to an effect of anticoagulation.

Besides the anticoagulation strategy, other factors may influence the occurrence of thromboembolic events, such as ethnicity: Liao et al. showed that Europeans have a significantly higher incidence of VTE compared with Maori, Pacific Island and Asian populations [33]. Moreover, this observation has been described in individuals of different ethnicities living within the same geographical location. In our opinion it is therefore important to conduct analyses in different locations to identify other possible confounders.

### Limitations

Our study has several limitations. It is a retrospective and single-center study comprising only a small number of 31 patients. In Switzerland, the first peak of new COVID-19 patients was reached in April 2020, and numbers decreased substantially at the beginning of May 2020. Extending our analysis over a longer period would therefore not have added many new cases. Although we predefined a set of coagulation analyses in order to facilitate a standardized coagulation monitoring this was not universally adopted and not always executed as repetitive determinations during the course of the disease. To avoid a potential bias of patients with multiple repetitive measurements, we only entered the mean value per week and patient into the figures. At the beginning of the COVID pandemia little was known about the associated coagulopathy. At that time, the standardized coagulation monitoring in our hospital included routine parameters (D-Dimers, PT/Quick, TT, fibrinogen and PLT count). Other parameters of interest (such as proinflammatory cytokines, markers of the fibrinolysis, or markers of the anticoagulation system) were determined infrequently and on a case-by-case basis, which limits their availability for analysis. Further studies are necessary to investigate these markers in more detail. Finally, our patients were not a homogenous collective. Most were severely ill patients treated in the ICU, but others in the ward or as outpatients. Comparison was limited by the small number of outpatients (n = 4) and ward patients (n = 5). Therefore, we performed a sensitivity analysis for ICU patients only.

## Conclusion

We observe coagulation alterations in COVID-19 patients indicating a significant hypercoagulability. These are present despite antithrombotic treatment, and peak around week 3–4 of the disease. ICU patients may even have higher D-dimer levels. This could be an argument for a prolongation of thromboprophylaxis and for an increased dose in critically ill patients or patients with additional prothrombotic risk factors.

## Supporting information

S1 Material (DOCX)

## Abbreviations

ARDS: acute respiratory distress syndrome
CI: Confidence interval
COVID-19: Corona virus disease 2019
CRP: C-reactive protein
ECMO: extracorporeal membrane oxygenation
ICU: intensive care unit
LMWH: low-molecular-weight heparin
SARS-CoV-2: Severe acute respiratory syndrome Corona virus 2
SSH: Swiss Society of Hematology
UFH: unfractionated heparin
