# Supplementary material for: Coronavirus-induced coagulopathy during the course of disease
Source: PLoS One. 2020 Dec 17;15(12):e0243409. doi: 10.1371/journal.pone.0243409 (PMC7745968; doi:10.1371/journal.pone.0243409)
Supplement: S1 Material — (DOCX) [file pone.0243409.s001.docx]

**Coronavirus induced coagulopathy during the course of disease**

S1 Material

| **S1 Table:** Impact of anticoagulation on thrombin time. We observed an almost linear increase of the thrombin time over time (figure 1) and performed a linear regression model entering unfractioned heparin (UFH), argatroban and stay as additional explanatory variables. The increase of the thrombin time is to the most extent attributed to the effect of anticoagulation. | | | | |
| --- | --- | --- | --- | --- |
| Intercept | | 6.5 | -22.1 to 34.7 | 0.66 |
| Day of illness | | 0.64 | 0.2 to 1.1 | 0.004 |
| UFH | prophylactic | 25.2 | -5.0 to 53.9 | 0.11 |
|  | therapeutic | 41.6 | 12.2 to 69.7 | 0.007 |
| Argatroban | therapeutic | 98.8 | 66.0 to 132.1 | <0.0001 |
| Stay | ward | 18.0 | -19.5 to 56.8 | 0.37 |
|  | ICU | -18.3 | -57.7 to 22.7 | 0.39 |

**Additional sensitivity analyses including ICU patients only:**

As a sensitivity analysis, we excluded the 9 patients that were treated as outpatients or on the ward and conducted the analyses with ICU patients only. In the following we analyze the impact of time of illness on different coagulation parameters with linear regression models. Detailed course of each parameter during the disease is presented subsequently in supplementary figure 1.

**D-dimers:** tobit regression coefficient 0.43, 95% CI 0.29 to 0.57; p<0.0001

**Fibrinogen:** tobit regression coefficient -0.04, 95% CI -0.05 to -0.02; p<0.0001

**Platetelets:** linear regression coefficient 0.78, 95% CI -1.16 to 2.7; p=0.43

**Quick:** tobit regression coefficient 0.06, 95% CI -0.03 to 0.15; p=0.17

**Anti-Xa activity:** linear regression coefficient 0, 95% CI 0 to 0; p=0.12

**Thrombin time:** linear regression coefficient 0.7, 95% CI 0.19 to 1.22; p=0.007

**S1 Fig:** Weekly boxplots of D-dimer, PT/Quick, fibrinogen, anti-Xa activity, thrombin time, and platelet count during the course of disease of ICU patients only. To avoid a potential bias of patients with multiple repetitive measurements, only the mean value per week and patient was entered in the figures.

**
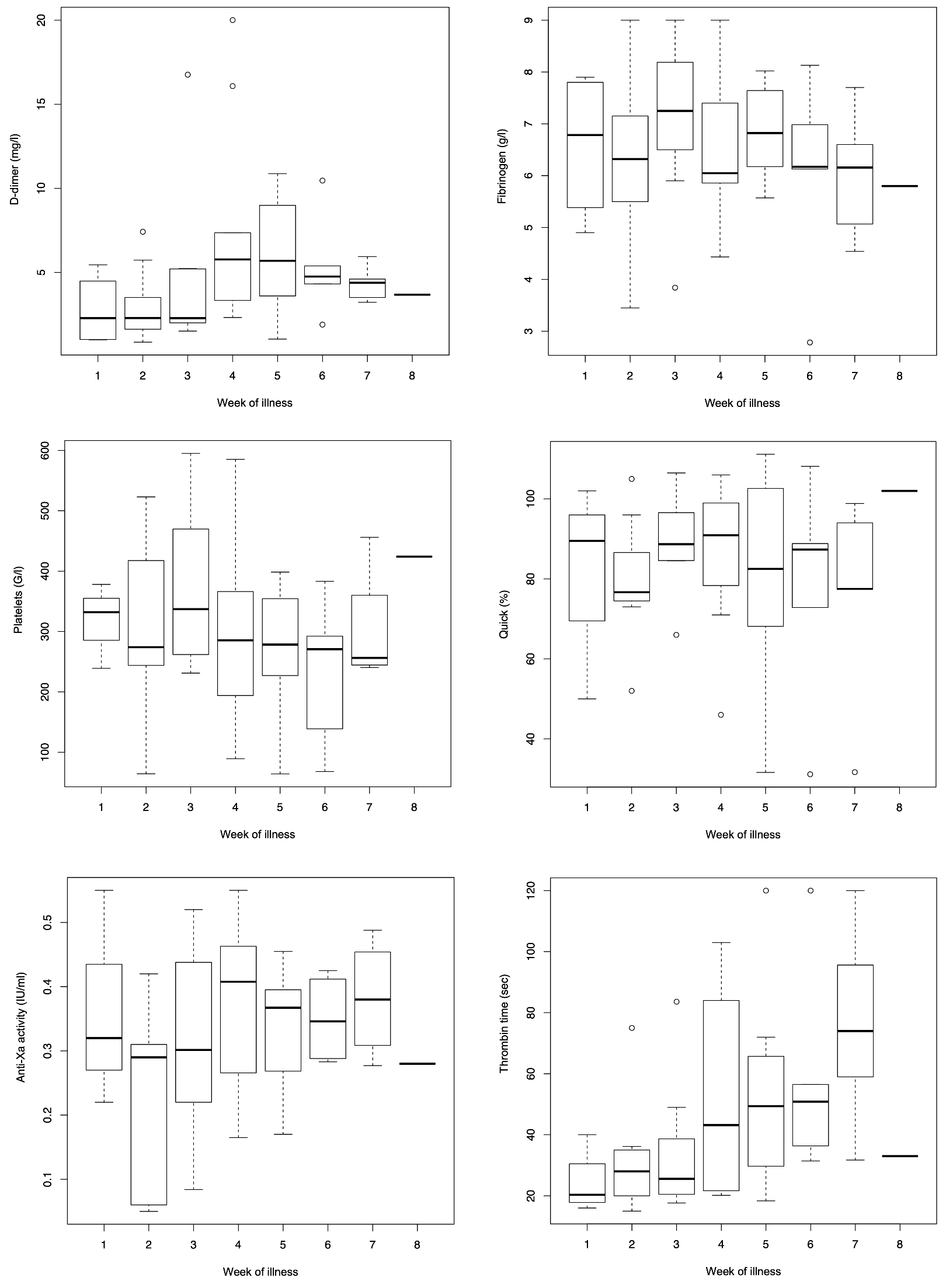
**
